# Fast Approximation for Sparse Coding with Applications to Object Recognition

**DOI:** 10.3390/s21041442

**Published:** 2021-02-19

**Authors:** Zhenzhen Sun, Yuanlong Yu

**Affiliations:** The College of Mathematics and Computer Science, Fuzhou University, Fuzhou 350116, China; zhenzhen_sun@foxmail.com

**Keywords:** sparse coding, fast approximation, homotopy iterative hard thresholding, object recognition

## Abstract

Sparse Coding (SC) has been widely studied and shown its superiority in the fields of signal processing, statistics, and machine learning. However, due to the high computational cost of the optimization algorithms required to compute the sparse feature, the applicability of SC to real-time object recognition tasks is limited. Many deep neural networks have been constructed to low fast estimate the sparse feature with the help of a large number of training samples, which is not suitable for small-scale datasets. Therefore, this work presents a simple and efficient fast approximation method for SC, in which a special single-hidden-layer neural network (SLNNs) is constructed to perform the approximation task, and the optimal sparse features of training samples exactly computed by sparse coding algorithm are used as ground truth to train the SLNNs. After training, the proposed SLNNs can quickly estimate sparse features for testing samples. Ten benchmark data sets taken from UCI databases and two face image datasets are used for experiment, and the low root mean square error (RMSE) results between the approximated sparse features and the optimal ones have verified the approximation performance of this proposed method. Furthermore, the recognition results demonstrate that the proposed method can effectively reduce the computational time of testing process while maintaining the recognition performance, and outperforms several state-of-the-art fast approximation sparse coding methods, as well as the exact sparse coding algorithms.

## 1. Introduction

Object recognition is a fundamental problem in machine learning, and has been widely researched for many years. The performance of object recognition methods largely relies on feature representation. Traditional methods used handcrafted features to represent objects, i.e., scale-invariant feature transform (SIFT) [1], histograms of oriented gradients (HOG) [2], etc. Inspired by biological finding [3,4], learning sparse representation is more beneficial for object recognition, because mapping features from low-dimensional space to a high-dimensional space makes the features more likely to be linearly separable. Therefore, many sparse coding (SC) algorithms have been proposed to learn a good sparse representation for natural signals [5,6,7].

In general, SC is the problem of reconstructing input signal using a linear combination of an over-complete dictionary with sparse coefficients, i.e., for an observed signal $x\in R^{p}$ an over-complete dictionary $D\in R^{p\times K}\left(p\ll K\right)$, SC aims to find a representation $\alpha \in R^{K}$ to reconstruct x by using only a small number of atoms chosen from D. The problem of SC is formulated as

(1) $$\begin{array}{c} \underset{\alpha }{\min:}||x-{D\alpha ||}_{2}^{2}+{\lambda ||\alpha ||}_{0}, \end{array}$$

where the $l_{0}$-norm is defined as the number of non-zero elements of α, and λ is the regularization factor. Several optimization algorithms have been proposed for the numerical solution of (1). However, the high computational cost induced by these optimization algorithms is a major drawback for real-time applications, especially when a large-sized dictionary is used.

To get rid of this problem, many works focusing on fast approximation for sparse coding have been proposed. Kavukcuoglu et al. [8] proposed a method named Predictive Sparse Decomposition (PSD) that used a non-linear regressor to approximate the sparse feature, and applied this method to objection recognition. However, the predictor is simplistic and produces crude approximation, and the regressor training procedure is somewhat time-consuming because of the gradient descent training method. Recently, deep learning showed its widespread success on many inference problems, which provides another way to design fast approximation methods for sparse coding algorithms. The idea is first proposed by Gegor et al. [9] who constructed two deep learning networks to approximate the iterative soft thresholding and coordinate descent algorithms, leading to the so-called LISTA and LCoD methods, respectively. LISTA showed its superiority on calculation and approximation, and many recent variants of LISTA have been proposed for miscellaneous applications, see [10,11] for some examples. Inspired by [9], many fast approximation sparse coding methods based on deep learning have been proposed and shown their effectiveness on unfolding the corresponding sparse coding algorithms, i.e., LAMP [12], LVAMP [12], etc.

Though these methods perform well in large-scale datasets, there are three defects. First, they are not suitable for small-scale datasets, in which the number of training samples is far less then ten thousand. The performance of deep neural network is sensitive to the scale of training data, when the number of training samples is small, the deep network model is over-parameterized and may result in over-fitting. Second, deep networks involve lots of hyper-parameters, whose training requires large computational and storage resources because of the gradient-based back-propagation method, and is easy to get stuck in a local optimal solution. Last but not least, each deep network architecture is designed only for the corresponding sparse coding algorithm that cannot be generalized to other algorithms. Therefore, the extendibility of these methods are limited.

To solve the problems mentioned above, a simple and effective fast approximation sparse coding method is proposed for small-scale datasets object recognition task in this paper. Differing from the deep learning-based methods, a special single-hidden-layer neural network (SLNNs) is constructed to perform the approximation task, and the training process of this SLNNs can be easily implemented by the least squared method. The proposed method includes two steps. In the first step, the optimal sparse features of training samples are exactly computed by sparse coding algorithm (in this paper, the homotopy iterative hard thresholding (HIHT) algorithm [13] is used), and in the second step the optimal sparse features are used as ground truth to train the especially constructed SLNNs. After training, the input layer and hidden layer of this SLNNs can be used to implement the nonlinear feature mapping from the input space to sparse feature space, which only involves simple inner product calculation with a non-linear activation function. Therefore, the sparse features of new samples can be estimated quickly. Ten benchmark datasets taken from UCI databases and two face image datasets are used to validate the proposed method, and the root mean square error (RMSE) results on testing data have verified the approximation performance of this proposed method. Furthermore, the approximated sparse features have been applied to object recognition task, and the recognition results demonstrate that this proposed approximation sparse coding method is beneficial for object recognition in terms of recognition accuracy and testing time.

The main contributions of this paper can be concluded as

1. A fast approximation sparse coding method is proposed for small-scale datasets object recognition task, which can quickly estimate the sparse features for testing samples.
2. A special SLNNs architecture has been constructed to perform the approximation task, whose parameters can be optimized easily by the least squared method, avoiding the multifarious procedure induced by the gradient-based back-propagation training.
3. Experiment results on ten benchmark UCI datasets and two face image datasets show that our approach is more effective than current state-of-the-art deep learning-based fast approximation sparse coding methods both in RMSE, recognition accuracy and testing time.

The remainder of this paper is organized as follows. Section 2 briefly reviews the sparse coding algorithms and fast approximation sparse coding methods. Section 3 details the proposed method. Section 4 describes implementation details and presents experimental results. Finally, conclusions are given in Section 5.

## 2. Related Work

### 2.1. Sparse Coding Algorithms

As described in Section 1, the problem of SC can be formulated as problem (1). However, problem (1) is NP-hard, which is difficult to be solved. There are three common methods for approximations/relaxations of this problem: (1) iterative greedy algorithms [14,15,16]; (2) $l_{1}$-norm convex relaxation methods (which are called basis pursuit) [17]; (3) $l_{p}$-norm (0<p<1) relaxation methods [18,19,20,21,22]. Among these methods, BP has been studied more widely, in which the $l_{0}$ norm is replaced by $l_{1}$ norm to make a convex relaxation for the problem (1), i.e.,

(2) $$\begin{array}{c} \underset{\alpha }{\min:}||x-{D\alpha ||}_{2}^{2}+{\lambda ||\alpha ||}_{1}, \end{array}$$

where the $l_{1}$-norm is defined as the sum of absolute values of all elements of α.

BP methods were proven to give the same solutions to (1) when the dictionary satisfies the Restricted Isometry Property (RIP) condition [23,24]. Many research works focusing on efficiently solving problem (2) have been proposed, [25] provides a comprehensive review of five representative algorithms, namely *Gradient Projection* (GP) [26,27], *Homotopy* [28,29], *Iterative Soft Shrinkage-Thresholding Algorithm* (ISTA) [30,31,32], *Proximal Gradient* (PG) [33,34], and *Augmented Lagrange Multiplier* (ALM) [35]. Among these algorithms, ISTA is the most popular algorithm, and lots of heuristic strategies have been proposed to reduce the computational time of ISTA, i.e., TwIST [36], FISTA [33], etc. Recently, a kind of pathwise coordinate optimization method called PICASSO [37,38,39] has been proposed to solve the $l_{p}$ (0<p≤1) least squared problem, which showed superior empirical performance compared with other state-of-the-art sparse coding algorithms mentioned above.

Although satisfactory results can be achieved by using the approximation/relaxation methods, the $l_{0}$-norm is more desirable from the sparsity perspective. In recent years, researchers have attempted to solve problem (1) directly, with iterative hard thresholding (IHT) [13,40,41] being the most popular method. The IHT methods have strong theoretical guarantees, and the extensive experimental results show that the IHT methods can improve the sparse representation reconstruction results.

### 2.2. Fast Approximation for Sparse Coding

The sparse coding algorithms mentioned in Section 2.1 involve a lot of iterative operations, which induces high computational cost and prohibits them from real-time applications. To get rid of this problem, some research focusing on fast approximation for sparse coding was proposed. Kavukcuoglu et al. [8] proposed the PSD method to approximate sparse coding algorithms using a non-linear regressor. In inspired by this, Chalasani et al. [42] extended PSD to estimate convolutional sparse features. However, the approximation performance of non-linear regressor is limited. As the development of deep learning, some researchers have constructed deep networks to solve the fast approximation sparse coding problem. Given a large set of training examples ${\left\{\left(x_{i},\alpha_{i}\right)\right\}}_{i=1}^{N}$, a many-layer neural network is optimized to minimize the reconstruction mean squared error between network outputs and ${\left\{\alpha_{i}\right\}}_{i=1}^{N}$. After training, the approximation of sparse representation for a new signal $x_{new}$ can be quickly predicted by the deep network. The idea is first proposed by Gregor et al. [9] who constructed two deep learning networks to approximate the iterative soft thresholding and coordinate descent algorithms, leading to the so-called LISTA and LCoD methods, respectively. Inspired by [9], Xin et al. [43] translated the iterative hard thresholding algorithm into a deep learning framework. Borgerding et al. [12] proposed two deep neural-network architectures to unfold the approximate message passing (AMP) algorithm [44] and “vector AMP” (VAMP) algorithm [45] respectively, namely LAMP and LVAMP. In [46], the authors proposed a deep learning framework for the approximation of sparse representation of a signal with the aid of a correlated signal, the so-called side information. The learned deep networks perform steps similar to those implemented by corresponding sparse coding algorithms; however, the trained network can reduce the computational cost when calculating the sparse representation of new samples effectively, which is critical in large-scale data settings and real-time applications.

## 3. Materials and Methods

### 3.1. Homotopy Iterative Hard Thresholding Algorithm

The homotopy iterative hard thresholding (HIHT) [13] is an extension of IHT for the $l_{0}$-norm regularized problem

(3) $$\begin{array}{c} \underset{\alpha }{\min:}\theta_{\lambda }\left(\alpha \right)=f\left(\alpha \right)+{\lambda ||\alpha ||}_{0}, \end{array}$$

where $f\left(\alpha \right)=\frac{1}{2}||x-{D\alpha ||}_{2}^{2}$ is a differentiable convex function, whose gradient ∇f(α) satisfies the Lipschitz continuous condition with parameter $L_{f}>0$. Therefore, f(α) can be approximately iteratively updated by the projected gradient method

(4) $$\alpha^{k+1}=argmin\;f\left(\alpha^{k}\right)+{▽f(\alpha^{k})}^{T}\left(\alpha -\alpha^{k}\right)+\frac{L}{2}||\alpha -\alpha^{k}{\left|\right|}_{2}^{2},$$

where L≥0 is a constant, which should satisfies the condition of $L⩾L_{f}$.

Adding ${\lambda ||\alpha ||}_{0}$ into both side of (4), the solution of (3) can be obtained by iteratively solving the subproblem

(5) $$\begin{array}{cc} \alpha^{k+1} & =arg\min\;p_{L,\lambda }\left(\alpha^{k},\alpha \right)\\ & =f\left(\alpha^{k}\right)+\nabla f{\left(\alpha^{k}\right)}^{T}\left(\alpha -\alpha^{k}\right)+\frac{L}{2}||\alpha -\alpha^{k}{\left|\right|}_{2}^{2}+{\lambda ||\alpha ||}_{0}. \end{array}$$

The optimization of (5) is the same as follows (by removing or adding some constant items which are independent on α):

(6) $$\min_{\alpha }\frac{L}{2}\left[||\alpha -\left(\alpha^{k}-\frac{1}{L}\nabla f\left(\alpha^{k}\right)\right){\left|\right|}_{2}^{2}+\frac{2\lambda }{L}{\left||\alpha |\right|}_{0}\right].$$

If denote

(7) $$T_{L}\left(\alpha^{k+1}\right)=\underset{\alpha }{arg\min}\;\;p_{L,\lambda }\left(\alpha^{k},\alpha \right),$$

then the closed form solution of $T_{L}\left(\alpha^{k+1}\right)$ is given by the following lemma.

**Lemma** **1.** *[32,41] The solution $T_{L}\left(\alpha^{k+1}\right)$ of (7) is give by*

(8) $${\left[T_{L}\left(\alpha^{k+1}\right)\right]}_{i}=\left\{\begin{array}{ccc} {\left[s_{L}\left(\alpha^{k}\right)\right]}_{i}, & \; & if\;{\left[s_{L}\left(\alpha^{k}\right)\right]}_{i}^{2}>\frac{2\lambda }{L};\\ 0, & \; & if\;{\left[s_{L}\left(\alpha^{k}\right)\right]}_{i}^{2}\leq \frac{2\lambda }{L}. \end{array}\right.$$

*where $s_{L}\left(\alpha \right)=\alpha -\frac{1}{L}\nabla f\left(\alpha \right)$, and ${\left[.\right]}_{i}$ refers to the i-th element of a vector.*

In (8), the parameter *L* needs to be tuned. The upper bound on Lipschitz constant $L_{f}$ is unknown or may not be easily calculated, thus we use the line search method to search *L* as suggested in [41] until the objective value descends.

***Homotopy Strategy***: many works [13,26,39] have verified that the sparse coding approaches benefit from a good starting point. Therefore, we use a recursive process automatically tunes regularization factor λ. This process begins from a large initial value $\lambda^{0}$. At the end of each λ-tuning iterations indexed by *k*, an optimal solution $\alpha^{k}$ is obtained given $\lambda^{k}$. Then λ is updated as $\lambda^{k+1}=\rho \lambda^{k}$, where ρ∈[0,1], and $\alpha^{k}$ is used as the initial solution for the next iteration k+1. The process stops once λ is small enough (given a positive lower-bound target, the stop condition is $\lambda_{k}\leq \lambda_{target}$). An outline of HIHT algorithm is described as Algorithm 1.
**Algorithm 1**$\left\{\alpha^{★},L^{★}\right\}\leftarrow HIHT\left(L_{0},\lambda_{0},\alpha^{0}\right)$ **(Input:)**
$L_{0},\lambda_{0},\alpha^{0},D,L_{min},L_{max};//L_{0}\in \left[L_{min},L_{max}\right]$ **(Output:)**
$\alpha^{★},L^{★}$; initialize ρ∈(0,1),η>0,γ>1,ϵ>0,k←0; **repeat**  i←0;  $\alpha^{k,0}=\alpha^{k}$;  $L_{k,0}\leftarrow L_{k}$;  **repeat**    *An L-tuning iteration indexed by i*   $\alpha^{k,i+1}\leftarrow T_{L_{k,i}}\left(\alpha^{k,i}\right)$;   **while**
$\theta_{\lambda_{k}}\left(\alpha^{k,i}\right)-\theta_{\lambda_{k}}\left(\alpha^{k,i+1}\right)<\frac{\eta }{2}\left|\right|\alpha^{k,i}-\alpha^{k,i+1}{\left|\right|}^{2}$**do**
     $L_{k,i}\leftarrow min\left\{\gamma L_{k,i},L_{max}\right\}$;     $\alpha^{k,i+1}\leftarrow T_{L_{k,i}}\left(\alpha^{k,i}\right)$;   **end while**   $L_{k,i+1}\leftarrow L_{k,i}$;   i←i+1;   **until**
$\left|\right|\alpha^{k,i}-\alpha^{k,i+1}{\left|\right|}_{2}^{2}\leq \epsilon$
   $\alpha^{k+1}\leftarrow \alpha^{k,i}$;   $L_{k+1}\leftarrow L_{k,i}$.   $\lambda_{k+1}\leftarrow \rho \lambda_{k}$;   k←k+1;
 **until**
$\lambda_{k+1}\leq \lambda_{target}$
 $\alpha^{★}\leftarrow \alpha^{k}$; $L^{★}\leftarrow L_{k}$.

### 3.2. Proposed Method

**Figure 1:**
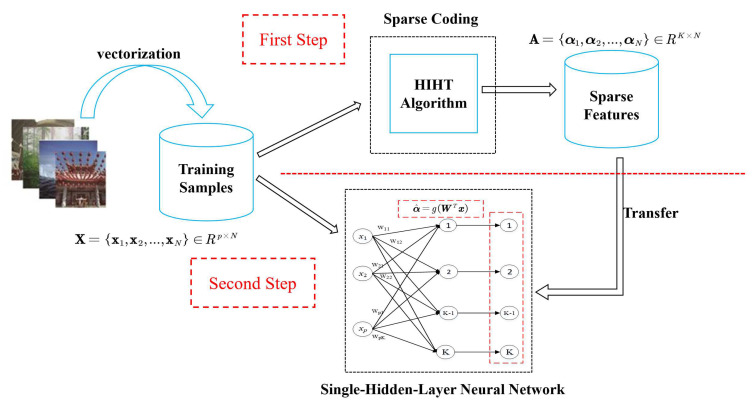
The schematic diagram of this proposed method.

Figure 1 illustrates the schematic diagram of this proposed method. As it can be seen, for the given training dataset $X=\left\{x_{1},x_{2},...,x_{N}\right\}\in R^{p*N}$ and the over-complete dictionary D, the HIHT algorithm described in Section 3.1 is used to calculate the optimal sparse features $A=\left\{\alpha_{1},\alpha_{2},...,\alpha_{N}\right\}\in R^{K*N}$ of training data in the first step. After that, these optimal sparse features are used to train the SLNNs in second step.

As Figure 1 shows, the architecture of the neural network consists of an input layer, a feature layer and an output layer. The number of hidden neurons is the same as that of output neurons, which is set as the dimension of the sparse feature. Each hidden neuron is only connected to its corresponding output neuron with weight 1. Our goal is to obtain a optimal input weights $\hat{W}$ to make the outputs of hidden layer as equal to A as possible, that is

(9) $$||g\left({\hat{W}}^{T}X\right)-A{\left|\right|}_{F}\to 0,$$

where g(.) refers to a non-linear activation function.

There are two strategies to optimize the input weights W:

(1) If the activation function is known, we chose *tanh* function as the activation function, where g(x)=tanh(x). We firstly calculate arctanh(A), and denote the result as Z, that is

(10) Z=arctanh(A),

then we formulate the objective function of the SLNNs as

(11) $$\begin{array}{c} \mathrm{Minimize}:\;\frac{1}{2}∥Z-W^{T}{X∥}_{F}^{2}+\frac{C_{1}}{2}{∥W∥}_{F}^{2}, \end{array}$$

where constant $C_{1}$ refers to the regularization factor used to control the trade-off between the smoothness of the mapping function and the closeness to Z.

By setting the derivative of (11) with respect to W to zero and solve this equality, then the optimal solution of W is obtained as follows:

(12) $$\hat{W}={\left(\frac{I}{C_{1}}+XX^{T}\right)}^{-1}XZ^{T}.$$

In addition, the sparse feature of a testing sample $x_{test}$ can be quickly estimated as

(13) $${\hat{\alpha }}_{test}=tanh\left({\left({\left(\frac{I}{C_{1}}+XX^{T}\right)}^{-1}XZ^{T}\right)}^{T}x_{test}\right).$$

(2) If the activation function is unknown, a kernel trick based on Mercer’s condition can be used to calculate the approximated sparse feature of testing data $x_{test}$ directly instead of training the weights W,

(14) $${\hat{\alpha }}_{test}=g\left(W^{T}*x_{\mathrm{test}}\right)=Ker\left(x_{\mathrm{test}},X\right){\left(\frac{I}{C_{1}}+\Omega_{train}\right)}^{-1}A,$$

where $\Omega_{train}==Ker\left(X,X\right)$ and Ker stands for the kernel function.

In this proposed method, Gaussian function is used as the kernel function Ker:

(15) $$Ker\left(x_{1},x_{2}\right)=exp\left(-\frac{\left|\right|x_{1}-x_{2}{\left|\right|}^{2}}{\sigma^{2}}\right),$$

where σ denotes the standard deviation of the Gaussian function.

## 4. Results and Discussion

### 4.1. Data Sets Description

Ten benchmark datasets taken from *UCI Machine Learning Repository* [47] and two image datasets: the Extended YaleB [48] and the AR dataset [49], are used to validate the proposed method. The ten UCI datasets include 5 binary-classification cases and 5 multi-classification cases. The details of these datasets are shown in Table 1. In this table, column “Random Perm” shows whether the training and testing data are randomly assigned or not. In the experiments, $\frac{2}{3}$ of samples per class are randomly selected for training, and the rest samples are responsible for testing if “Random Perm” is Yes.

**Figure 2:**
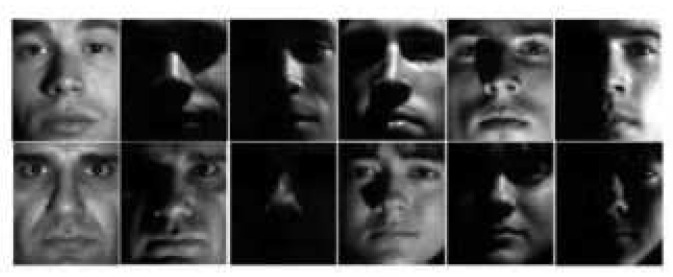
Extended YaleB.

The extended YaleB dataset [48] contains 38 different people with 2414 frontal face images, and each class has about 64 samples. This dataset is challenging from varying expressions and illumination conditions, see Figure 2 for some examples. The random face feature descriptor generated in [7] is used as raw feature, in which a cropped image with 192×168 pixels was projected onto a 504-dimensional vector by a random normal distributed matrix. In the experiment, 50% of samples per class are randomly selected for training and the rest are responsible for testing.

**Figure 3:**
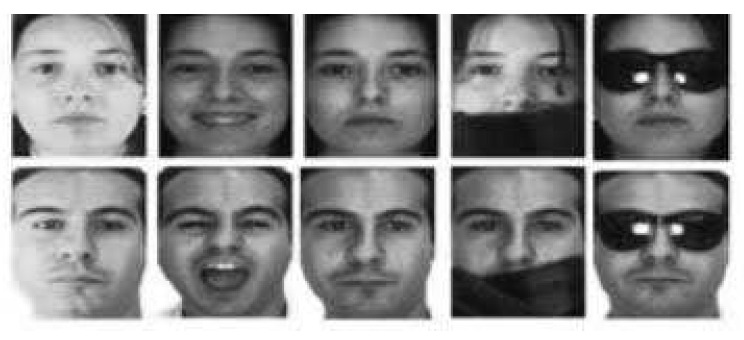
AR Face.

The AR face dataset contains over 126 people with more than 4000 face images. There are 26 images per person taken during two different sessions. The images have large variations in terms of disguise, facial expressions, and illumination conditions. A few samples from the AR dataset are shown in Figure 3 for illustration. A subset of 2600 images pertaining to 50 males and 50 females objects are used for experiment. For each object, 20 samples are randomly chosen for training and the rest for testing. The images with 165×120 pixels were projected onto a 540-dimensional vector by using a random projection matrix.

### 4.2. Implementation Details

The experiments are mainly divided into two parts: (1) The RMSE between the approximated sparse features and the optimal features of testing data is calculated to verify the approximation performance of this proposed method, and the results of several state-of-the-art fast approximation sparse coding methods are also reported for comparison. (2) Classification experiments are implemented to validate the recognition performance of the approximated sparse features estimated by the proposed SLNNs. The compared methods can be categorized as follows: (a) Different representation learning methods: ELM [50] with random feature mapping, and ScELM [51] with optimal sparse features computed by HIHT; (b) Different fast approximation sparse coding methods: PSD [8], LISTA [9], LAMP [12], and LVAMP [12], detailed descriptions to these methods are provided in Section 2.

Implementations of ELM, ScELM, PSD, and this proposed method are based on Matlab codes and others are based on Python. A random normal distributed matrix is used as the dictionary in each sparse coding algorithm, and the number of atoms or hidden nodes *K* is set to 100 if the dimension of dataset is less than 100, otherwise 1000. The parameter $C_{1}$ is searched for in the grid of $\left\{2^{-25},2^{-20},...,2^{25}\right\}$, and the σ is searched for in {100,200,...,1000}. The number of hidden layers of LISTA, LAMP, and LVAMP are set as 6, 5, and 4, respectively, if not stated otherwise. Other parameters are default as the authors suggested. For the randomly training-testing assigned datasets, ten repeated trials are carried out in the following experiments, and the average result and standard deviation are recorded.

In object recognition experiments, the trained network of each method is used to compute the approximated sparse features for training and testing samples, and the approximated sparse features are used as the input of the classifier. The ridge regression model is used as the classifier in our experiments, whose objective function is

(16) $$\begin{array}{c} \mathrm{Minimize}:\;\frac{1}{2}∥Y-\beta^{T}\hat{A}{∥}_{F}^{2}+\frac{C_{2}}{2}{∥\beta ∥}_{F}^{2}, \end{array}$$

where Y is the label matrix of training data X, and β is the weights of the classifier model. For a testing sample $x_{test}$, the predicted label for it is calculated as

(17) $$\begin{array}{c} identity\left(x_{test}\right)=argmax_{i}\left(\beta^{T}{\hat{\alpha }}_{test}\right). \end{array}$$

The hyper-parameter of the classifier $C_{2}$ is searched for in the grid of $\left\{2^{-25},2^{-20},...,2^{25}\right\}$, and a value with best validation accuracy is selected. We compare our method with others in terms of recognition accuracy and testing time, where the recognition accuracy is defined as the ratio of the number of correctly classified testing samples to that of all testing samples, and the testing time refers to the total spending time of testing samples’ feature calculation and classification.

A standard PC is used in our experiments and its hardware configuration as follows:

1. CPU: Intel(R) Pentium(R) CPU G2030 @3.40GHz;
2. Memory: 32.00GB;
3. Graphics Processing Unit (GPU): None.

### 4.3. Root Mean Square Error Results

For testing data $X_{test}$, whose optimal sparse features computing by sparse coding algorithm is denoted as $A_{test}$, and the approximated sparse features computing by the fast approximation method is denoted as ${\hat{A}}_{test}$, the RMSE between $A_{test}$ and ${\hat{A}}_{test}$ is defined as

(18) $$RMSE\left(A_{test},{\hat{A}}_{test}\right)=\sqrt{\frac{1}{N_{test}K}\left|\right|A_{test}-{\hat{A}}_{test}{\left|\right|}_{F}^{2}},$$

where $N_{test}$ denotes the amount of testing samples.

Some UCI datasets are used in this experiment, and we reported the results of our method, LISTA, LAMP and LVAMP to compare their approximation performance, Table 2 shows the results. As it can be seen from this table, our approach can achieve a lower RMSE result than other methods on the most datasets, which indicates that the approximated sparse features estimated by our approach are more closer to the optimal ones than that estimated by the compared methods. For the Glass dataset, our method has achieved a significant improvement, and for LiverDisorders, though the result of our approach is not the best one, it is very close to the best one.

### 4.4. Objection Recognition Results

#### 4.4.1. The Evaluation of HIHT

The existing literature on sparse coding only compared different sparse coding algorithms in terms of reconstruction error and convergence speed, but did not compare their classification performance when applying these algorithms in object recognition. To show why this paper uses the HIHT algorithm to compute the optimal sparse features, we implemented some experiments to validate the superiority of HIHT compared with several state-of-the-art sparse coding algorithms when used in object recognition. The compared methods include IHT, homotopy GPSR (HGPSR) [26], PGH [34], and PICASSO [39].

(1) *Effectiveness on Object Recognition*: the binary-classification datasets listed in Table 1 are used in this experiment. Firstly, the sparse coding algorithms are used to compute sparse features for the experimental datasets using the same dictionary, and the measure of *cross entropy* is used to show how different the sparse features are between class 1 and class 2. A higher value means that the sparse features computed by corresponding algorithm are more discriminative and more beneficial for object recognition. The measure of *cross entropy* is estimated as follows: we accumulate a histogram $h(\alpha_{k}|v)$ along feature dimensions over all sparse features $\alpha_{k}$ that belongs to the same class *v*(v∈{1,2}), then normalize the histogram as the probability p(v) of class *v*, the *cross entropy* between class 1 and class 2 is estimated as

(19) $$cross\;entropy\left(p\left(1\right),p\left(2\right)\right)=-\sum_{k=1}^{K}p_{k}\left(1\right)\log\frac{1}{p_{k}\left(2\right)},$$

where $p_{k}\left(v\right)$ is the p-th element of the probability p(v).

Table 3 shows the *cross entropy* results. It can be seen that the HIHT algorithm can achieve the best result on the most datasets than the other four algorithms. It indicated that the sparse features computed by HIHT can distinguish different classes more effectively, which is more useful for classification, especially when a simple linear classifier is used.

Subsequently, we use these sparse coding algorithms to compute the optimal sparse features of training data to train the proposed SLNNs, and compare the final recognition results, which is shown in Table 4. From this table it can be seen that these sparse coding algorithms can achieve similar classification performance on most datasets when used in the proposed method, while HIHT outperforms the other three algorithms in some datasets (i.e., Glass and Vehicle) significantly. From the view of standard deviation, the results show that the optimal sparse features computed by HIHT are more robust to classification than other algorithms.

(2) *Parameter Sensitivity*: In HIHT algorithm, different values of the regularization factor $\lambda_{target}$ and dictionary D will product different sparse features, which will cause the proposed method to estimate different approximated sparse features and influence final recognition result. In this experiment, the sensitivities of $\lambda_{target}$ and D in final recognition performance are verified, and the two face image datasets are used for testing.

**Figure 4:**
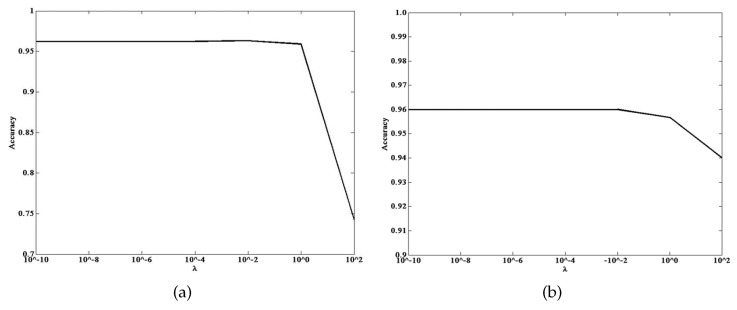
The influence of the target value $\lambda_{target}$ of HIHT on the recognition performance. (**a**) Extended YaleB. (**b**) AR Face.

Firstly, the influence of $\lambda_{target}$ is investigated. By fixing other parameters (i.e., dictionary, parameters of the classifier), $\lambda_{target}$ is searched for in the grid of $\left\{10^{-10},10^{-8},...,10^{2}\right\}$, and the corresponding recognition accuracy is recorded. From the results in Figure 4, we can conclude that the final recognition result is not very sensitive to the $\lambda_{target}$, so it is no need to spend much time turning $\lambda_{target}$ when uses HIHT to compute the optimal sparse features in this proposed method.

Subsequently, we investigate the influence of D. An unsupervised learned dictionary by the Lagrangian dual method [52] is used to compare with a random dictionary generated by normal distribution. The number of iterations in dictionary learning is set as 5, and 10 times with random selection of training and testing data are repeated, the average accuracy is recorded for comparison. As Table 5 shows, the final recognition accuracy achieved by using learned dictionary are close to that by using random dictionary. However, the computational time of optimal sparse features calculation with dictionary learning is five times (equal to the number of iterations) that with the random dictionary. Thus, in the following experiments we use random dictionary to compute optimal sparse features in HIHT algorithm.

#### 4.4.2. Evaluation on UCI Datasets

The average recognition accuracies on UCI datasets are listed in Table 6 and Table 7 presents the testing time. From these two tables we can conclude that the proposed approach outperforms other methods in terms of accuracy and testing time simultaneously. For most datasets, the approximated sparse features estimated by our approach can obtain the highest accuracy, and is approximately 100 times faster than ScELM (exact sparse coding algorithm), especially in high-dimensional datasets. Compared with other approximation sparse coding methods, our approach can achieve higher recognition accuracy with simpler network training, and the testing time of the proposed method and PSD are much less than LISTA, LAMP and LVAMP. It is worth noting that the performances of activation function tanh and kernel function of this approach are similar, but kernel function outperforms tanh when the dataset is a litter complex, (i.e., Satimage, Madelon), which will be confirmed in next experiments.

**Figure 5:**
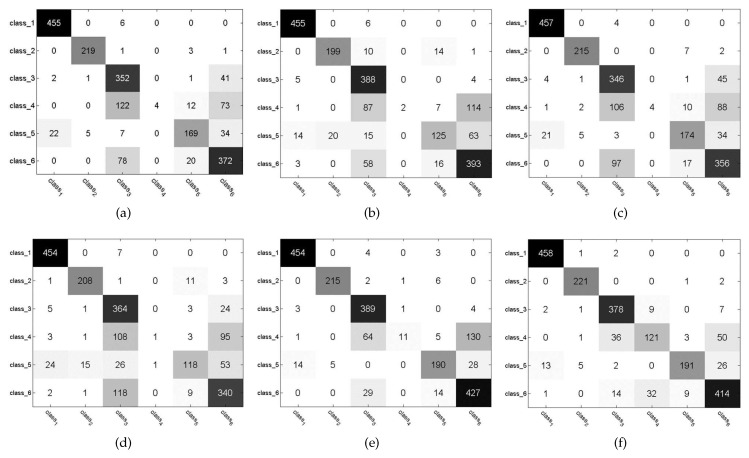
Confusion matrices on Satimage dataset. (**a**) PSD. (**b**) LISTA. (**c**) LAMP. (**d**) LVAMP. (**e**) Tanh. (**f**) Kernel.

**Figure 6:**
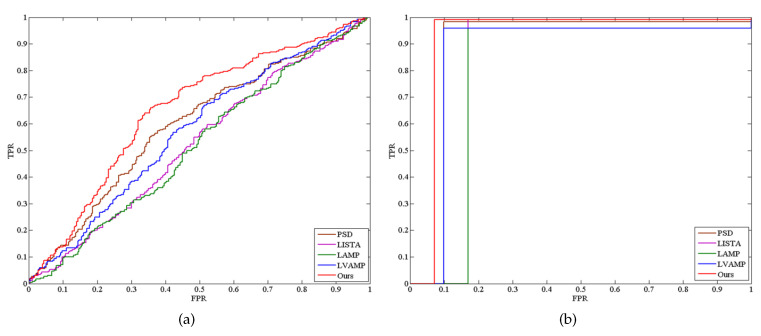
The receiver operating characteristic (ROC) curves of the approximate methods. (**a**) Madelon. (**b**) Breast.

Figure 5 (The Tanh and Kernel mean the tanh version and kernel version of our method, respectively.) shows the confusion matrices obtained by this proposed method, PSD, LISTA, LAMP and LVAMP on Satimage dataset, in which the kernel version of this proposed method achieved a much better recognition result than others. It can be seen from this figure that all methods almost fail to correctly classify the test samples of class 4 except the kernel version of our method. It indicates that the features computed by this proposed method are more discriminative than that of other approximation methods. Figure 6 shows two examples of the receiver operating characteristic (ROC) curves of the approximation methods, where the red lines report the performance of our approach. It is clear that the Areas Under ROC curves (AUC) of our approach is much higher than others.

#### 4.4.3. Evaluation on Extended YaleB Dataset

Table 8 lists the recognition accuracies and testing time on Extended YaleB dataset, in which the famous sparse representation-based face recognition algorithm SRC [53] and collaborative representation-based classification (CRC) [54] are also used for comparison. Furthermore, a result obtained by raw features is set as the error bar (denoted as Baseline), and we set different number of hidden layers (denoted as *T*) for LISTA to show its influence on object recognition performance. As Table 8 shows, all methods beat the Baseline, indicating the benefit of feature learning. The kernel version of this proposed method obtains the best result with the value of 98.33%, and is 1.89% higher than the second one. In testing process, the proposed method is approximately 21 times faster than the deep learning-based approximation methods, and 182 times faster than SRC, also much faster than CRC. For LISTA, if the number of layers is small (T=2), the recognition performance will degrade much, and as the number of layers increases, the recognition results tend to be stable. Thus, the recognition performance is somewhat sensitive to the number of layers of deep network.

**Figure 7:**
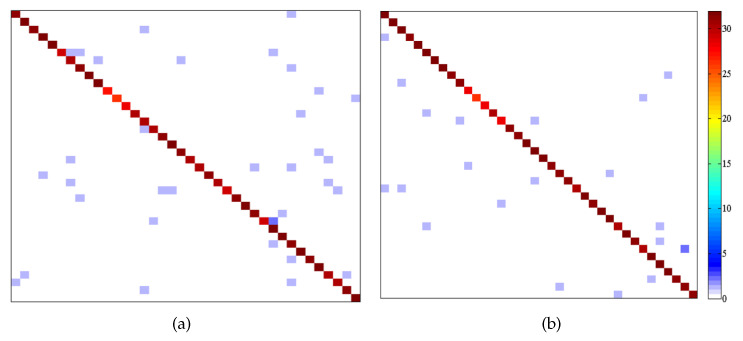
Patterns of confusion on Extended YaleB. (**a**) Tanh. (**b**) Kernel.

Figure 7 shows the patterns of confusion across classes obtained by this proposed method, in which coordinates in X-axis and Y-axis represent 38 face classes. Color at coordinates (x,y) represents the number of test samples whose ground truth are *x* while machine’s output labels are *y*. From this figure it can be seen that our approach shows fewer points in the non-diagonal region (i.e., fewer false positives and false negatives), indicting that the proposed method can classify most testing samples correctly.

#### 4.4.4. Evaluation on AR Dataset

For the AR dataset, a protocol (e.g., only five training samples per class or all training samples are used) is established in our experiments, and the corresponding results are list in Table 9. As we can see, the kernel version of this proposed method achieves the best result in both cases. In addition, the tanh version of this method gets comparable result with LAMP and ELM, but still better than SRC when all training samples were used. In terms of testing time, the proposed method is approximately 12 times faster than the deep learning-based approximation methods, 300 times faster than SRC, and 24 times faster than CRC. It is worth noting that the computational speed of kernel version is a little slower than that of tanh version, since it needs to compute the kernel matrix between testing samples and training samples, while it is still much faster than the deep learning-based approximation methods.

**Figure 8:**
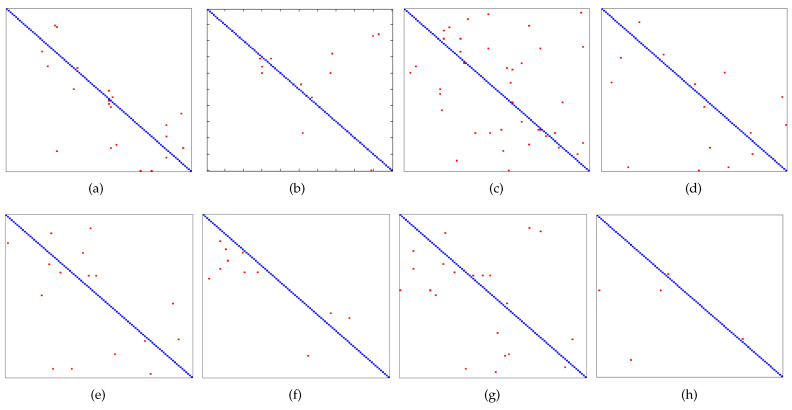
Patterns of confusion on AR dataset. (**a**) SRC. (**b**) CRC. (**c**) ScELM. (**d**) PSD. (**e**) LISTA. (**f**) LAMP. (**g**) LVAMP. (**h**) Kernel.

We use a confusion matrix to give the detailed evaluation at the class-level. Figure 8 shows the results, in which coordinates in *x*- and *y*-axis denote 100 face classes. Red point with coordinates (x,y) represents the misclassified test samples. It can be seen from this figure that this proposed method shows rare points in the non-diagonal region than other methods, indicating that this proposed method performs better than other methods in object recognition.

**Figure 9:**
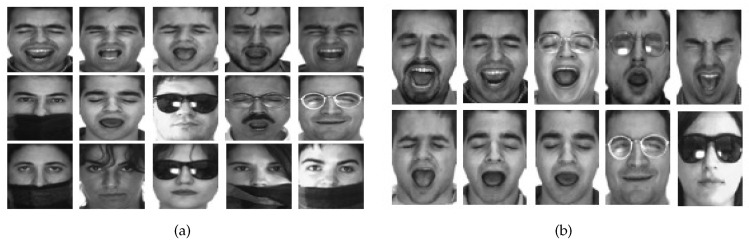
All misclassified images produced by (**a**) LAMP and (**b**) Kernel.

To give an intuitive illustration, Figure 9 shows all misclassified images obtained by LAMP method (which achieves the second best result) and this proposed method (kernel version). It can be seen that images with exaggerated facial expressions is the main reason causing misclassification for both methods. Another interesting point can be seen that most images with facial “disguises” are misclassified by LAMP method while they are correctly recognized by this proposed method. It indicates that the approximate sparse features estimated by this proposed method is robustness to facial occlusion or corruption than LAMP.

## 5. Conclusions

This paper proposes a simple fast approximation sparse coding method for small-scale datasets object recognition task, in which the optimal sparse features of training data computed by HIHT algorithm are used as ground truth to train a succinct and special SLNNs, thus make the representation learning in object recognition task more practical and efficient. Extensive experimental results on publicly available datasets show that this approach outperforms the compared approximation methods in terms of approximation performance, recognition accuracy and computational time. The high recognition and computational efficiency makes the proposed method very promising for real-time applications. Moreover, experimental results have demonstrated that this proposed method is robust to parameters on recognition performance, that make it more practical. Future work includes supervised sparse coding algorithms and autonomously finding an over-complete dictionary.

## Figures and Tables

**Table 1:** UCI Data sets Used in Our Experiments.

Datasets	Training	Testing	Features	Classes	Random Perm
Australian Credit	459	231	14	2	Yes
Diabetes	511	257	8	2	Yes
Glass	140	74	9	6	Yes
Image segmentation	1540	770	19	7	Yes
LiverDisorders	229	116	6	2	Yes
Madelon	2000	600	500	2	No
Satimage	4435	2000	36	6	No
Vehicle	562	284	18	4	Yes
Wine	118	60	13	3	Yes
Wisconsin Breast Cancer	379	190	30	2	Yes

**Table 2:** The root mean square error of compared methods on UCI datasets (bold one represents the best result).

Datasets	LISTA	LAMP	LVAMP	Ours	
				Tanh	Kernel
Australian Credit	0.0418	0.0614	0.0497	0.0591	**0.0391**
Diabetes	0.0460	0.0356	0.0446	0.0494	**0.0350**
Glass	0.0527	0.0436	0.0550	**0.0111**	0.0144
Image segmentation	0.0324	0.0406	0.0462	0.0375	**0.0317**
LiverDisorders	0.0440	**0.0403**	0.0435	0.0476	0.0597
Vehicle	0.0267	0.0442	0.0496	0.0141	**0.0122**
Wine	0.0415	0.0394	0.0462	0.0149	**0.0094**
Wisconsin Breast Cancer	0.0295	0.0551	0.0427	0.0222	**0.0179**

**Table 3:** Cross entropy of sparse features between different classes.

Datasets	HGPSR	IHT	PGH	PICASSO	HIHT
Austrain Credit	6.840	6.785	6.933	6.299	**7.268**
Diabetes	4.381	6.164	3.899	4.118	**6.410**
LiverDisorders	3.087	5.461	3.267	5.894	**6.346**
Madelon	8.040	9.335	8.076	9.280	**9.680**
Wisconsin Breast Cancer	4.652	**6.256**	4.643	5.632	5.878

**Table 4:** Recognition Results of the Proposed Method Using different sparse coding algorithms.

Datasets	HGPSR	IHT	PICASSO	HIHT
Australian Credit	85.59 ± 1.83	85.87 ± 1.87	85.67 ± 1.87	86.08 ± 1.73
Diabetes	76.06 ± 1.96	76.73 ± 1.68	74.96 ± 2.28	76.99 ± 1.66
Glass	60.97 ± 4.83	61.64 ± 3.38	64.59 ± 4.63	66.60 ± 3.20
Image segmentation	90.08 ± 1.02	90.81 ± 0.97	90.91 ± 0.89	91.90 ± 0.73
LiverDisorders	68.59 ± 3.62	72.34 ± 4.27	69.57 ± 3.96	73.52 ± 2.56
Madelon	59.67	60.88	58.33	60.67
Satimage	82.12	83.37	83.00	84.30
Vehicle	78.46 ± 2.47	78.68 ± 2.07	76.20 ± 1.81	81.00 ± 1.51
Wine	96.80 ± 1.92	98.53 ± 1.47	97.33 ± 1.81	98.56 ± 1.07
Wisconsin Breast Cancer	93.87 ± 1.68	95.31 ± 1.09	96.00 ± 2.89	95.92 ± 1.81

**Table 5:** Comparison of final recognition performance with or without dictionary learning in HIHT algorithm.

Datasets	Learned Dictionary	Random Dictionary
YaleB	95.95 ± 0.65	96.34 ± 0.69
AR	96.63 ± 0.77	97.37 ± 0.39

**Table 6:** The average accuracy of compared methods on UCI datasets (red is the best result and blue is the second one).

Datasets	ELM	ScELM	PSD	LISTA	LAMP	LVAMP	Ours	
							Tanh	Kernel
Australian Credit	86.13	83.52	86.22	81.97	80.26	-	86.08	86.15
Diabetes	65.32	69.26	65.01	73.98	68.19	74.59	76.99	75.14
Glass	57.30	62.38	63.46	67.50	60.50	61.75	66.60	67.23
Image segmentation	77.32	88.77	89.30	88.13	87.39	90.63	91.90	91.86
LiverDisorders	67.45	65.52	69.17	70.34	73.39	66.10	73.52	74.25
Madelon	58.17	59.17	59.35	51.67	50.92	56.93	60.67	64.83
Satimage	71.70	80.25	78.55	78.10	77.60	74.25	84.30	89.15
Vehicle	73.99	75.30	78.85	74.22	78.61	72.83	81.00	80.56
Wine	95.13	94.00	95.00	93.25	96.83	94.29	98.56	98.00
Wisconsin Breast Cancer	87.54	94.40	94.80	93.23	91.56	93.75	95.92	96.48

**Table 7:** The testing time of compared methods on UCI datasets

Datasets	ELM	ScELM	PSD	LISTA	LAMP	LVAMP	Ours	
							Tanh	Kernel
Australian Credit	0.0079	0.6696	$2.006\times 10^{-4}$	0.0728	0.0811	0.0728	$3.181\times 10^{-4}$	0.0033
Diabetes	0.0136	1.7844	$6.704\times 10^{-5}$	0.0756	0.1332	0.0927	$3.536\times 10^{-4}$	0.0057
Glass	0.0013	0.9932	$5.282\times 10^{-5}$	0.0527	0.0714	0.0505	$1.276\times 10^{-4}$	$6.061\times 10^{-4}$
Image segmentation	0.0029	22.226	$2.258\times 10^{-4}$	0.1833	0.4511	0.4998	$9.128\times 10^{-4}$	0.0365
LiverDisorders	0.0011	0.7055	$6.198\times 10^{-5}$	0.0797	0.2499	0.5070	$2.052\times 10^{-4}$	$9.227\times 10^{-4}$
Madelon	0.0494	148.59	0.0062	1.2150	1.2560	0.9656	0.0293	0.073
Satimage	0.0134	39.218	$7.130\times 10^{-4}$	0.4272	0.3601	0.3287	0.0028	0.3018
Vehicle	0.0015	1.2062	$1.013\times 10^{-4}$	0.1249	0.3214	0.0991	$4.392\times 10^{-4}$	0.0047
Wine	$7.232\times 10^{-7}$	0.2457	$5.687\times 10^{-5}$	0.1042	0.0783	0.0556	$4.392\times 10^{-4}$	$4.291\times 10^{-4}$
Wisconsin Breast Cancer	0.0021	2.7363	$2.086e\times 10^{-4}$	0.0625	0.0929	0.0731	$3.099\times 10^{-4}$	0.0134

**Table 8:** Average Recognition Accuracy with Random-Face Features on the Extended YaleB Database.

Method	Accuracy (%)	Time (s)
Baseline	91.54 ± 1.14	0.0009
SRC	96.27 ± 0.64	16.1676
CRC	96.82 ± 0.58	1.5006
ELM	96.44 ± 0.60	0.0256
ScELM	92.43 ± 0.93	168.3158
PSD	93.01 ± 0.69	0.057
LISTA (T = 2)	92.19 ± 1.21	2.0129
LISTA (T = 6)	95.16 ± 1.18	2.2535
LISTA (T = 10)	95.34 ± 0.82	4.6385
LAMP	94.07 ± 0.72	2.0119
LVAMP	94.62 ± 1.12	1.900
Tanh	96.34 ± 0.69	0.0256
kernel	**98.33 ± 0.40**	0.0888

**Table 9:** Average Recognition Accuracy with Random-Face Features on the AR Face Database. The four column is the result when only 5 training samples per class are used.

Method	Accuracy (%)	Time (s)	Accuracy (%)
Baseline	93.00 ± 0.84	0.0011	78.67
SRC	95.42 ± 0.59	27.1421	84.00
CRC	96.92 ± 0.76	1.9682	84.83
ELM	97.05 ± 0.78	0.0178	83.67
ScELM	94.50 ± 1.09	62.1872	73.50
PSD	96.80 ± 0.66	0.0305	77.17
LISTA (T=3)	95.57 ± 0.85	0.8888	-
LISTA (T=6)	96.47 ± 0.61	1.2335	80.33
LISTA (T=8)	96.80 ± 0.73	3.1065	-
LAMP	97.37 ± 0.59	1.0505	81.67
LVAMP	95.30 ± 0.97	1.0314	84.50
Tanh	97.37 ± 0.39	0.0189	85.33
Kernel	**98.40 ± 0.46**	0.0815	**86.67**

## Data Availability

Publicly available datasets were analyzed in this study. These datasets can be found here: http://archive.ics.uci.edu/ml/index.php.
